# Applying a gender lens on *human papillomavirus* infection: cervical cancer screening, HPV DNA testing, and HPV vaccination

**DOI:** 10.1186/1475-9276-12-14

**Published:** 2013-02-08

**Authors:** Ivan Branković, Petra Verdonk, Ineke Klinge

**Affiliations:** 1 Institute for Public Health Genomics, Department of Genetics and Cell Biology, FHML, School CAPHRI, Maastricht University, PO Box 616, MD 6200, Maastricht, The Netherlands; 2 Department of Medical Humanities, EMGO, Institute for Health and Care Research, School of Medical Science, VU University Medical Center, Amsterdam, The Netherlands; 3 Department of Health, Ethics and Society, FHML, School CAPHRI, Maastricht University, Maastricht, The Netherlands

**Keywords:** Gender, Human papillomavirus, HPV, HPV vaccine, Cervical cancer, Pap smear, HPV DNA testing, Intersectionality

## Abstract

**Background:**

Our aim is to provide a state-of-the-art overview of knowledge on sex (biological) and gender (sociocultural) aspects of *Human papillomavirus* (HPV) and cervical cancer for educational purposes. Considerable disparities exist in cervical cancer incidences between different subgroups of women. We provide an outline on the crucial issues and debates based on the recent literature published in leading gender medicine journals. Intersectionality was applied in order to help categorise the knowledge.

**Methods:**

Key terms (HPV, cervical cancer) were screened in Gender Medicine, Journal of Women’s Health and Women & Health from January 2005-June 2012. Additional searches were conducted for topics insufficiently mentioned, such as HPV vaccination of boys. In total, 71 publications were included (56 original papers, four reviews, six reports, three commentaries, one editorial and one policy statement).

**Results:**

Research reveals complexity in the way various subgroups of women adhere to cervical screening. Less educated women, older women, uninsured women, homeless women, migrant women facing language barriers, women who have sex with women and obese women participate in Pap smears less frequently. A series of barriers can act to impede decisions to vaccinate against HPV.

**Conclusions:**

Both male and female controlled preventive methods and treatment measures should be developed in order to tackle HPV infection and different strategies are needed for different subgroups. A substantial discussion and research on alternative methods of prevention was and is lacking. In future research, sex and gender aspects of HPV-related diseases of boys and men as well as subgroup differences in HPV risk need to be addressed.

## Introduction

Sexually Transmitted Infections (STIs) are a formidable public health issue. Many sex and gender differences exist in epidemiology, etiology, diagnosis, treatment and consequences of STIs. There is a clear distinction made between the two – the term ‘sex’ is reserved for hormonal, chromosomal or any other features stemming from person’s biology. Gender, on the other hand, pertains to sociocultural concepts of femininity and masculinity, which can vary between cultures [1]. For instance, besides biological differences related to the reproductive roles of men and women that cause differences (such as women’s higher biological vulnerability for STIs), gender differences occur because women have less power over sexual situations than men [2]. Gender aspects interact with biological sex differences in infectious diseases [3].

*Human papillomavirus* (HPV) infection is one of the most common STIs, although it is not solely transmitted sexually, and oncogenic subtypes are associated with cervical cancer, as well as cancers of the head and neck, anal tumors, penile cancers, and cancers of the vulva and vagina [4]. Infection with High-Risk (HR) HPV strain is necessary but insufficient for cervical cancer to develop. Currently known co-factors associated with cervical cancer development are cigarette smoking, alcohol consumption, micronutrients deficiency in fruits and vegetables, prolonged use of oral contraception, multiparity, uncircumcised male partner, low socioeconomic status (SES), infection with HIV/AIDS or other STIs including herpes simplex and *Chlamydia trachomatis*[5,6]. However, other additional factors may also hinder the immune system in shielding the organism from these infections. Violence compromises the body’s ability to counter the infection as well via stress behaviours such as smoking or alcohol consumption [7]. However, the prevalence of stressful life events or self-reported stress are not always found to be associated with higher risk for cervical disease [8]. Cervical cancer is also associated with different types of violence: childhood sexual abuse (CSA), intimate partner violence, or forced sexual experiences [7]. A consequence of CSA is engaging in sexual risky behaviour such as intercourse with casual partners, not using condoms, earlier age of first consensual intercourse, failing to discuss intercourse in advance, having sex with a partner who injects drugs, or having an HIV positive partner [9]. Studies show that CSA is reported by twice as many women than men (prevalence approximately 30% vs. 15%) and more often by subgroups such as pregnant adolescents, men who have sex with men, lesbian and bisexual women, women in psychiatric care, drug users or persons who tested positive for HIV [9-11].

Developments such as the HPV vaccines and the implementation of HPV DNA testing for diagnostic purposes have led to an increase in publications on HPV and heated public debate. Gender analysis enables us to elucidate different patterns of interaction of gender with race, class, and other factors [12], which can help us understand how gender relates to HPV infection, HPV vaccination and DNA testing.

Men and women cannot be divided into two homogenous groups, and people’s group memberships such as gender, age, ethnic background, sexual orientation, religious affiliation (both assigned and self-identified) redefine each other. Intersectionality is an approach that addresses the way gender and other social identities affect life, and refers to mutually constitutive relationships that influence each other [13-15]. An intersectionality approach is based in social justice, and aims to address processes of inclusion and exclusion in order to address issues of marginalised groups [16]. The approach explains how a single focus on gender, race, or any factor on its own makes it impossible to examine the manner in which social forces and locations interact and relate to each other in order to give shape to the unique mosaic of human experiences. The elements are fluid and flexible, and a subgroup at risk lies at the intersection – the place all relevant elements interact. Gender is therefore essential, but should not be decoupled from other categories, for this would disable the contextual analysis [15]. In this literature review we apply intersectionality in order to understand the role gender plays in HPV. Thus, we put a gender lens on HPV and summarise recent publications from three leading journals in gender medicine.

With an eye for intersectionality, we aim to categorise recently published knowledge on cervical cancer screening, HPV DNA testing and HPV vaccination. According to leading journals in gender medicine, what is state-of-the-art in regards to gender knowledge about HPV and cervical cancer?

## Materials and methods

The study is funded by the European Union Erasmus Curriculum Development Project Gender Medicine EUGiM (2009–2011). State-of-the-art education material in gender medicine was developed and used during a postgraduate summer school in Gender Medicine, which was piloted in Berlin, Germany in September 2010 and in Sassari, Italy in September 2011.

In order to obtain a good and timely overview in gender and HPV for education purposes, we searched for recent relevant literature in three renowned and leading gender medicine journals: Women & Health, Gender Medicine, and Journal of Women’s Health. We opted for the choice of these journals due to the fact that relevant gender-specific publications are often not retrieved by either conventional searches or the use of filters. Moreover, authors who are insufficiently familiar with the field often misuse the terms ‘sex’ and ‘gender’; for instance by applying the term ‘gender’ even in cases of strictly biological differences [17]. The selected journals, however, cover a broad range of sex and gender-related aspects and tend to the appropriate use of terminology and indexing. Women & Health publishes papers on the physical and psychological health of women, including environmental factors and prevention, historical overviews on women’s health and health policy research. Gender Medicine explicitly publishes papers that use sex and/or gender approaches in their design. The Journal of Women’s Health takes a multidisciplinary approach to health and illness. Hence, a broad overview of recent research that would include gender aspects of HPV was anticipated. We searched volumes from January 2005-June 2012 and read title and abstracts. This timeframe covers studies on the launch and implementation of the two HPV vaccines and developments in HPV DNA testing. We included papers if the abstracts mentioned HPV, cervical cancer and related issues (risk factors, behaviours, screening and prevention). Additional hand searches were performed in order to provide sufficient information on the HPV vaccine debate and the vaccination of boys. All types of publications were included. After reading the text, we organised the literature based on whether it reports on Pap smears, HPV DNA testing or HPV vaccination. In order to avoid excessive referencing, we reduced the number of cited articles in the text while still focusing on the major factors presented in the literature.

In total, 71 papers were included in this review, out of which 56 original papers, four reviews, six reports, three commentaries, one editorial and one policy statement. The overview of included papers per year is provided in Table 1.

**Table 1:** Numbers of included papers per each year with reference numbers in square brackets

**Publication year**	**Cervical cancer screening**	**HPV DNA testing**	**HPV vaccination**
2005	**2**[18,19]	**3**[20-22]	**0**
2006	**3**[23-25]	**4**[26-29]	**2**[30,31]
2007	**2**[32,33]	**1**[34]	**7**[5,7,35-39]
2008	**5**[40-44]	**0**	**1**[45]
2009	**12**[46-57]	**0**	**9**[46,58-65]
2010	**6**[66-71]	**0**	**3**[72-74]
2011	**2**[75,76]	**0**	**4**[77-80]
2012	**1**[81]	**1**[82]	**4**[83-86]
total	**33**	**8**	**30**

## Results

The following sections present main findings from the published research on cervical cancer screening, HPV DNA testing and HPV vaccines, with an additional focus on the debate over HPV vaccination and the issue of vaccinating the boys.

### Cervical cancer screening

Differences in HPV prevalence and cervical cancer screening rates between subgroups of women may exist due to a mix of patient characteristics, healthcare factors, and patient and physician attitudes. From 1995–2005 in Europe, cervical cancer incidence and mortality has declined, although in some countries it increases, such as Lithuania, Romania, and Bulgaria [6]. Globally, the Pap test is the principal means of detecting abnormalities, although organisation of screening programmes, coverage, eligibility criteria and recommended screening intervals differ. The Pap smear has become routine and reduced the incidence and mortality of cervical cancer [46], but cervical cancer still causes significant morbidity and mortality in developing countries. For instance in Nigeria, the prevalence of HR HPV and cytological abnormalities in women is 16.6% and consistent with other regions in Africa [40]. In American studies, Latinas were less likely to die of the disease than other groups [23]. Black women diagnosed with localised cervical cancer less likely had surgery, suggesting less optimal treatment [47]. In New Zealand, ethnic disparities in cancer survival are reported as well: Maori women were diagnosed more often with late stage cervical cancer, and had shorter survival, although excess mortality decreased over the years 1994–2005 [48]. Both SES and ethnic background are associated with poorer survival.

In many countries, screening programmes are set up for adult women at certain intervals. Overall, women who maintain on-schedule Pap tests appear to the generally healthier than women who do not obtain regular Pap smears [49]. In the US, women are advised to undergo annual screening or every 3 years if they had three consecutive normal Pap tests [20]. Free cancer screening programmes exist for low-income and uninsured women [41]. Different factors and their mutual interplay appear to play a role in cervical cancer screening rates. The overview of studies in which a number of relevant factors were observed, together with the explanation of the manner in which the factors affect the screening rates, is given in Table 2.

**Table 2:** Various factors influencing the participation rates of women in cervical cancer screening

**Factors**	**Main findings**
Knowledge	– a highly consistent factor contributing to higher participation of women in Pap screening [41,50,66]
SES	– low socio-economic status is associated with higher cervical cancer rates, lower Pap smear rates, and inadequate follow up [41,50,51]
	– women age 50+ with higher education are increasingly more up-to-date regarding screening services with each educational level [32]
Healthcare, access to healthcare, insurance	– not having health insurance is associated with not having a recent Pap test in southern US women [41]
	– universal healthcare appears to contribute to the reduction of socio-economic status related differences or differences in screening based on residential location [75]
	– an older study however showed that social factors discourage Australia's Indigenous women's use of and access to health services for screening, diagnosis and treatment of cervical cancer [24]
Age	– younger women age 19–26 exhibit more knowledge and participate more in preventive practices than women age 40–70 [81]
Marital status	– participation is higher in married women in Kuwait compared to unmarried women [50]
History of cervical infection, family history	– higher prevalence of ever having a Pap test is observed in women with either personal or family history of cancer [50]
Health expert’s willingness to give screening recommendation	– physician's recommendation is one of the strongest predictors of having had a Pap test [41]
Lifestyle	– smokers and obese persons adhere to Pap testing less frequently [49]

With regards to HPV prevention, screening and treatment, many subgroups of women are underserved such as women living in rural areas, lesbian women, older women, or women with low health literacy levels. Cancer screening programme satisfaction is a critical outcome for the healthcare system, and for patients with ethnic background, SES and health status play a role in patient satisfaction [42]. They often have to be addressed in a language not sufficiently familiar to them.

The findings on higher frequency of testing in African American women in the south of the US [41] may be an indicator that the racial gap in receiving Pap smears in the US is closing. The fact that non-Hispanic whites adhere less to Pap screening might be an outcome of years long campaigning within minority communities [33]. To date, experiences of maltreatment were informative of black people’s skepticism towards healthcare, such as side effects of HPV vaccine [87]. Within the Asian American community there are prominent differences, with the Korean subgroup showing the lowest screening rates. Japanese American women participate in cervical cancer screenings considerably less compared to other cancer screenings [67]. Chinese women in the US have lower screening rates mainly due to language barriers and lack of general knowledge, however education and interactions with a Chinese physician largely increased screening rates [68]. In China, women’s status is inferior to men’s, and women are more likely to live in poverty. SES affects Chinese women’s health care seeking behavior for self-reported genitourinary symptoms. Contrary to conventional wisdom and possibly due to more knowledge and awareness of stigma, Chinese women with high SES may be just as likely or even less likely to seek treatment for their symptoms [52].

Women who have sex with women (WSW) or lesbian women have lower screening rates even though HPV is transmittable between female sex partners and WSW often do also have (occasionally) sex with male partners [43,53]. WSW’s risk perceptions for HPV are lower than expected given the prevalence of abnormal Pap smears and HPV diagnosis, and thus, characterised by optimistic bias [43]. WSW may not seek out routine care by physicians, partly because they do not need prescriptions for contraception. Experiencing the healthcare system as being heterosexist evolves from the assumed heterosexuality evident in waiting rooms, health forms, and healthcare providers’ assumptions [44]. Fear of discrimination plays a role in delaying healthcare seeking [69]. In Canada, lesbian and bisexual women reported poorer health status on several health measures, but bisexual women negatively stood out. This group particularly appears to be not well served in Canadian healthcare [54], which was also reported in the US [55]. In the UK, lesbian women are more likely to avoid screening than heterosexual women, and more likely to have never attended screening than American lesbian women [18]. In the US, regular cervical cancer screening rates were equal among women partnering with men, women, or both [53]. Although a high percentage of lesbian women engage in sex with women and men, lesbian women may have a lower cervical cancer risk. However, lesbian women are more frequently obese and smoke more often, which may increase their cervical cancer risk [69]. Other subgroups of women at risk are presented in the following Table 3.

**Table 3:** Different subgroups of women at heightened risk from cervical cancer

**Subgroup**	**Main findings**
Perimenopausal women	- increased HPV prevalence [56]
	- both exogenous and endogenous hormones were associated with HPV infection [56]
Women over 55 years of age	- rarely initiate conversations on sexual matters [25]
	- physicians tend to initiate these discussions more with African American women [25]
Incarcerated women	- these women (but also incarcerated men), as well as those with a partner being released from prison are at risk due to their sexual behaviour [19,70]
	- more frequent history of abnormal Pap smears, particularly those exposed to violence [76], however they are also more receptive to prevention [70]
Women with disabilities	- may have lower screening rates due to difficulties associated with pelvic examinations[71]
	- Canadian women with traumatic spinal cord injury did not have lower screening rates, possibly due to frequent visits to physicians [71]
	- liquid cytology Pap smears are a reasonable alternative for screening this subgroup of women [57]

### HPV DNA testing

Current infections can be measured with highest sensitivity by HPV DNA testing, which can also be combined with Pap smears for optimising detection of high-grade cervical intraepithelial neoplasia [82]. Women with a negative HR HPV DNA test and a negative Pap test can extend their Pap screening interval [20,26,34]. Protocol redesign could be focused on those at high risk and be more cost-effective than existing protocols [27]. However, a few questions still remain. First, a Pap-plus-HPV test has higher sensitivity, but lower specificity, and thus raises the false-positive rate together with the true-positive rate which may result in over-treatment. Secondly, the high prevalence of transient infections among young women is high. And thirdly, a higher number of women with persistent infections but normal cervices will be detected. Therefore, a Pap-plus-HPV test may be more appropriate for women with borderline or mild Pap abnormalities, because a negative HPV DNA test could reassure women that their Pap test result is likely to be aberrant whereas treatment for women with a positive HPV DNA test may be started more rapidly [27]. In the United Kingdom, before introduction, HPV testing was perceived as an added value and would not negatively affect participation in Pap screening [21]. Informing women of their HPV status has both benefits and risks. A positive HPV test result may promote safer sexual practices; empower to adhere to Pap screening and follow-up; or engage in actions to prevent genital warts or cervical cancer. On the other hand, negative psychological side effects can occur such as stigma, blame and shame [21,28]. Self-collecting vaginal samples to test for HPV DNA seems feasible and counteracts barriers to screening attendance such as uncomfortable or anxiety-provoking pelvic examinations and contact with providers [22]. Self-collection of an HPV DNA test was highly acceptable among women because it was easy to perform, not painful, private, and could be conducted by the women themselves. However, the highest acceptability rates were found in subgroups with the highest screening rates already. More educational efforts are needed as many women were concerned whether they had done the self-collection test correctly [22].

In developing countries, where screening is often ineffective or under-utilised if existent at all, screening by female nurses appeared to be highly acceptable [29]. New screening methods such as HPV DNA testing, but also new visual inspection methods, provide opportunities for low-resource settings, decrease the number of visits of patients, and can be managed by mid-level healthcare providers [29]. New genetic technologies may increase our knowledge not only on pathogen genetics but also on host genetic factors as regards HPV. Individual genetic susceptibilities may exist with regards to HPV infection and progress into cervical cancer.

### HPV vaccine

A Pap smear detects abnormalities after they occur, thus pre-cancerous lesions may be missed. Therefore the discovery of the link between HPV and cervical cancer, and hence, the possibility of immunisation, enabled primary prevention. In sharp contrast with routinising Pap smears, the medical community, policymakers, and the public showed rapid attention to the HPV vaccines [46]. In 2006, the US Food and Drug Administration (FDA) approved of a noninfectious recombinant quadrivalent HPV vaccine (Gardasil) targeting HPV-16 and 18 as well as HPV-6 and 11 against genital warts for use in girls and women aged 9–26. In 2008, the second vaccine (Cervarix) targeting HPV-16 and 18 was marketed. In the US, the Center for Disease Control (CDC) and the American Medical Women’s Association recommend that women are vaccinated against HPV [30]. Young women are targeted for two reasons. First, immunological response is strongest in girls aged 10–15, and secondly, the vaccine is most efficacious in women who have not yet had sex [5]. The acceptability of the vaccine is decisive to its implementation [30] and insurance coverage predicts HPV vaccination, for instance among young, low-income, urban, predominantly black women. Also, abnormal Pap test in the past and normative beliefs that medical providers, parents, and others approve predicts HPV vaccination as well [58]. A study of young rural women revealed however that those with a reported history of an abnormal Pap test or of never having had a Pap test declined free HPV vaccination more frequently and vice versa. Higher refusal was also observed in women engaging in behaviours that increase the risk of HPV infection, e.g. mutual masturbation [77].

Low awareness of HPV exists among women and men across different age categories, geographical locations and racial/ethnic backgrounds [5,59,78]. Even in groups that demonstrate very high HPV awareness, such as young women at universities, the knowledge is often moderate. This particularly pertains to Latinas and women opposed to premarital sex [79]. In a study of the US girls aged 11–26 receiving their first injection, accurate knowledge on HPV and HPV vaccine increased with their age [83]. Hutson and colleagues [80] explain how the HPV knowledge gap is present across a broad age spectrum – in women aged 18–49. In their study of Appalachian women they describe how the absence of knowledge acts as a strong barrier to vaccination. The study revealed their greater concerns over vaccine's side effects rather than over potential promiscuity effect [80]. Latina mothers’ acceptability of the HPV vaccine for their children was generally high, even higher among HPV-positive mothers [60]. In Singapore however, women had low levels of awareness and incomplete knowledge of HPV, but acceptability of HPV vaccination was high, although acceptability to vaccinate their children was lower for HPV vaccination than for other preventable diseases [59].

In a survey study among family practitioners, general practitioners (GPs), and pediatricians, their agreement with professional recommendations was the most important variable determining intention to vaccinate young female patients [72]. Gynecologists and obstetricians would not offer vaccines mainly because of the costs and the belief that others should provide vaccines [61]. A recent CDC report on the US physicians' knowledge on the types of cancer that HPV vaccine is effective against [84] revealed their lower awareness that the vaccine presents not only cervical, but also vaginal, vulvar and anal cancer. Only around one quarter of respondents were aware of the effectiveness of the vaccine in preventing these other types of cancer. Health service providers working with American Indian/Alaska Native populations (who have a higher rate of cervical cancer incidence compared to non-Hispanic white women) often think that a pregnancy test should precede HPV vaccination. Also, they are more reluctant to vaccinate younger patients, which is of concern given the optimal age when the vaccine exerts maximum benefits [85].

Other barriers, such as costs to parents and difficulties getting adolescents to primary healthcare providers, are still unsolved in many countries. Lower vaccine initiation was associated with having parents with low incomes, having public insurance, and having fewer sexual partners [86].

### Debating HPV vaccines

Despite the enthusiastic reception of the HPV vaccines by women’s health advocates, women at risk, and public health officials, an intense debate has taken place [35]. A major concern raised is the vaccines’ effect on screening practices. Clinicians need to understand the difference between HPV vaccination to prevent cervical dysplasia and vaccinations for childhood infectious diseases, as effectiveness must be evaluated over decades [36]. Anti-vaccinationists questioned the vaccines’ safety and efficacy [45], and the assumption that there is one age at which all girls are negative for HPV fails to account for the girls who acquired HPV not through sexual contact, or for the 10-15% of sexually abused girls [36]. US conservatives framed the vaccine as ‘the promiscuity vaccine’ and feared that vaccinating preteen girls would sabotage their message of abstinence from sex before marriage by ‘disinhibition effects’, although such claims were countered by the CDC. Nevertheless, the debate has influenced public opinion about HPV vaccination and the percentage of parents in favour of mandatory HPV vaccination has declined. The relationship of the HPV vaccine with the evocation of a young/teenage women’s sexuality has had a particular effect which was not seen in other vaccines, for instance Hepatitis B [45,62].

Others claim that vaccination violates parents’ rights [5,45]. Intentions to vaccinate seem highest when the vaccine is presented as of little or no cost to women’s family and as preventing cancer, and when HPV was not described as an STI. Costs are thus a realistic barrier for intention to vaccinate and stigma still follows HPV [63]. In Australia, high levels of acceptance exists towards vaccination but reservations emerged when women understood the association between HPV and sexual activity [37]. In other countries, the debate is more to be framed as anti-vaccinationist and questions were raised about the vaccine’s long-term protection or validity in the ‘real world’. A systematic review on HPV vaccination shows that it is highly efficacious in preventing HPV infection and precancerous cervical disease, but long-term follow up to substantiate reductions in cancer incidence and mortality is needed in more representative populations of women [38]. Two RCTs to evaluate the effect of the quadrivalent vaccine in preventing cancerous lesions and genital warts showed sustained protection against low grade (I) lesions attributable to one of the four HPV types - 6, 11, 16 and 18 - and a substantial reduction of the disease burden 42 months follow-up [73]. The most frequently reported adverse effects are pain at the injection site (78%), bruising or discoloration (17%), swelling (14%), as well as fainting (15%). These reactions were reported more in younger girls than the older ones [83]. The vaccine is safe and well-tolerated, although side effects can only be ruled out at large numbers of vaccines [5]. A study using the Vaccine Adverse Event Reporting System to identify cervical cancer and carcinoma *in situ* among vaccinated women showed a few cases of cervical dysplasia and carcinoma *in situ*[74]. The women may have been exposed to HPV before vaccination, or to other HR strains. Moreover, vaccine failures may occur [74].

In Canada, other argumentations were heard. Women’s health advocates said there were too many unanswered questions and since cervical cancer in Canada has been declining, no cervical cancer epidemic exists to warrant the urgency of a vaccination programme. They warn not to overstate the relationship between HPV and cervical cancer because most HPV infections clear spontaneously [39]. Only 2-5% of all women with a cancerous HPV infection will develop cervix carcinoma within 12–15 years [4].

### Boys and HPV vaccination

Little attention is paid to whether boys should be vaccinated against HPV as well [64]. Boys are not targeted for vaccination, which is hardly debated. For instance, in half of the US newspaper articles on the HPV vaccine men were not mentioned as possible recipients [31]. But immunity of the population, or herd immunity, can only be reached with a gender-inclusive vaccination policy given the fact that men play an important role as STI carriers [64]. Another question is why there is so little knowledge available on male specific HPV-related disease to begin with. HPV seems associated with testicular cancer as well as with reduced sperm motility and infertility in men [65]. HPV-related disease such as oropharyngeal cancer, conjunctival squamous cancer, genital warts and anal and penile cancer does occur among some groups of men, such as men who have sex with men (MSM) and HIV positive men [36,65]. Proposals that boys should receive HPV vaccine to prevent cancer in women are far more common than claims about preventing penile and anal cancer in men [65].

## Discussion

In addition to biological sex differences [3], sociocultural gender differences, as well as their interaction at many levels, predict HPV risk and risk behaviour, healthcare access and screening rates, and consequences. In biomedical sex and sociocultural gender research, an intersectional approach is the next step forward [1]; it provides knowledge on how men and women’s different positions on aspects such as SES, ethnic background, or sexual orientation are interdependent and influence STI risk and health. According to Hankivsky et al. [88], intersectionality directs attention to health conditions that are more serious for certain groups of women and men. The relevance of sex and gender cannot be assumed a priori – other crucial aspects, such as SES, might be overshadowed by doing so [89]. Using pre-selected categories (for instance men and women) and treating them as homogenous and detached from other variables would be counterproductive, as they redefine each other [15]. Gaining insight into what happens within and between different groups takes research one step beyond subgroup analyses, which assumes categories such as gender or SES as independent categories. However, unequal social relations are incorporated in broad systems of historical inequalities which intersect, overlap, and reinforce each other to shape a person’s health status. Their connections to institutions such as healthcare, education, and law can be the target of intervention rather than individuals, in order to improve health [90]. We aimed to provide a comprehensive compendium of HPV-related health issues, diagnostics and prevention and present this knowledge by applying this approach. To our knowledge, there are no studies applying gender analysis coupled with intersectionality approach to this topic. By targeting the complexity of the intersections we aimed to shed light on particular subgroups which should be targeted when dealing with health disparities.

Our study points out several main findings. It shows that little knowledge of particularly men’s health in HPV seems available in gender medicine journals, despite the obvious relationship between gender and STIs and despite the definition of gender as a relational concept. Existing knowledge about men seems to focus on MSM, which has the potential to stigmatise MSM, and leaves men who have sex with women as well as those who have sex with men and women out of the picture. However, in every health condition and even more so in STIs, men and women’s health are intertwined. Less educated women, older women, uninsured women, homeless women, migrant women facing language barriers, WSW and obese women in most societies participate less often in Pap smears. Certain groups of women who were traditionally at a higher risk of cervical cancer (e.g. African American women) appear to be benefiting from community-based awareness-raising programmes, as a drop in prevalence can be observed. Other groups at increased risk, like homeless women, decline free Pap smears nevertheless. Thus, reasons for lower screening rates among some (sub)groups are evidently complex. Also, within those groups known to be at higher risk, for instance older women, we can observe the positive effect of education on screening participation. This bares implication for policy development and improvements, by helping to narrow down the focus of intervention to those less educated, therefore using the resources more effectively.

Our method yielded many papers on HPV and HPV related topics, mainly due to the launch of the HPV vaccine in this time period. Studies on awareness and attitudes in different (sub)groups are still frequent in journals with a gender focus. Since we chose to incorporate papers from three journals, we may have missed certain aspects in our overview. The men’s health movement advocates research in men’s health issues and in the past years several journals have been established in this field. Future research may focus on their scope in the field of STIs and HPV.

The European Commission requires attention for gender aspects in health research, and EU-funded researchers must address sex and gender research in their designs [90]. This study is but one example of why that ought to be an imperative. Our overview does provide input for a research agenda for the benefit of both men and women’s health. First, research on boys and men pertaining to HPV, both biomedical and gender research, needs to be accelerated. Furthermore, subgroups of men need attention, such as men with many sex partners, ageing men who use sildenafil, and also men who have sex with men and women. Lastly, research should also aim for a better understanding of how synergies of social factors contribute to both men and women’s HPV risk.

## Conclusions

As regards HPV prevention, screening, and treatment, many subgroups of women are underserved such as women living in rural areas, WSW, older women, or women with low health literacy levels. Often those at higher risk of cervical cancer tend to show lower awareness and knowledge regarding the HPV virus and HPV vaccine. In acquisition and transmission of HPV, gender relations are essential because of: physical and biological aspects; structural factors such as access to healthcare; social differences between men and women and unique interplays of social factors for each subgroup. Research on the role of host genetic susceptibilities may yield more insights on the risk factors in the near future. Various measures for preventing acquisition and transmission of STIs, such as male circumcision, need to be more seriously considered and with an open mind [91]. Female controlled methods (for instance female condoms, microbicides, and diaphragm) can be initiated by women themselves, which could make them a powerful resource for women to protect their own health [92,93]. However, these measures to reduce transmission of STIs are still studied less often and are still undergoing clinical studies [93]. In order to target STIs and HPV infection, both male and female controlled preventive and treatment measures need to be developed and different strategies are needed to reach subgroups of men and women. An open mind towards different strategies for prevention of HPV and a conceptualisation of gender as a relational and intersectional concept in which the health of men and women impact each other is of major importance.

## Competing interest

No competing financial interests exist.

## Authors’ contribution

All authors assume responsibility for the results of this manuscript. All authors read and approved the final manuscript.
